# Construction of an Extracellular Matrix–Related Risk Model to Analyze the Correlation Between Glioblastoma and Tumor Immunity

**DOI:** 10.1155/bmri/2004975

**Published:** 2025-03-10

**Authors:** Jian Li, Hong Pan, Yangyang Wang, Haixin Chen, Zhaopeng Song, Zheng Wang, Jinxing Li

**Affiliations:** ^1^Guangzhou University of Chinese Medicine, Guangzhou, Guangdong, China; ^2^Department of Neurosurgery, Linyi People's Hospital, Linyi, Shandong, China; ^3^Department of Ophthalmology, Shandong Provincial Hospital Affiliated to Shandong First Medical University, Jinan, Shandong, China; ^4^Department of Neurosurgery, Shandong Provincial Hospital Affiliated to Shandong First Medical University, Jinan, Shandong, China; ^5^Department of Neurosurgery, Liaocheng Traditional Chinese Medicine Hospital, Liaocheng, Shandong, China

**Keywords:** extracellular matrix, gene signature, glioblastoma multiforme, nomogram, prognosis

## Abstract

**Background:** Abnormalities in the extracellular matrix (ECM) have been shown to play a crucial role in promoting the formation, progression, and metastasis of glioblastoma multiforme (GBM). Therefore, our study is aimed at constructing a prognostic model based on ECM-related factors, to predict the prognosis of patients with GBM.

**Methods:** We employed single-sample gene set enrichment analysis (ssGSEA) to establish the ECM index of GBM. The identification of candidate genes was achieved by differential analysis conducted between ECM index groups, as well as through the utilization of weighted gene coexpression network analysis (WGCNA) and gene enrichment analysis. We conducted functional validation to confirm the significance of five biomarkers that were tested in the U251 cell line. The screening of prognostic genes was conducted using least absolute shrinkage and selection operator (LASSO) and univariate Cox analysis. The predictive relevance of the risk score model was assessed by using Kaplan–Meier (KM) survival curves in both The Cancer Genome Atlas (TCGA) and Chinese Glioma Genome Atlas (CGGA) cohorts. In addition, we conducted immunological studies, created and verified a nomogram, and constructed a network involving long noncoding RNA (lncRNA), microRNA (miRNA), and messenger RNA (mRNA).

**Results:** We identified 45 candidate genes by overlapping the 59 WGCNA core genes with the 855 differentially expressed genes (DEGs) between ECM index groups. These candidate genes were significantly enriched in 254 biological processes (BPs), 18 cellular components (CCs), 27 molecular functions (MFs), and 11 KEGG pathways. We identified a prognostic ECM-related five-gene signature using these candidate genes and constructed a risk model. Furthermore, we generated a nomogram model with excellent predictive ability. We also found significant differences between risk groups in six cell types and 29 immune checkpoints. Finally, we constructed a lncRNA–miRNA–mRNA network consisting of 27 lncRNAs, 73 miRNAs, and 5 model mRNAs.

**Conclusion:** Our study developed a prognostic model based on the ECM-related five-gene signature, which can serve as a valuable reference for the treatment and prophetic prediction of GBM.

## 1. Introduction

Glioblastoma multiforme (GBM) is an aggressive primary malignant tumor of the adult central nervous system (CNS) [1]. It has a low 5-year relative survival rate of 4%–5%, the weakest among all cancers, and a median overall survival (OS) of less than 15 months after treatment [2]. High-level ionizing radiation is the primary identified risk factor for GBM development [3]. The 2021 World Health Organization (WHO) classification categorizes GBM into IDH-wildtype GBM and IDH-mutant GBM [4]. IDH-wildtype GBM accounts for approximately 90% of cases, while IDH-mutant GBM mainly originates from low-grade diffuse gliomas and represents around 10% of cases [5]. Treatment options for GBM include surgical resection and chemotherapy using drugs like trimetazidine (TMZ), camptothecin, and bevacizumab. However, the overall prognosis for GBM patients remains poor despite these treatments [6–8]. Therefore, there is a critical need to identify biomarkers that can predict patient prognosis, which would not only guide treatment decisions but also advance our understanding of GBM's underlying mechanisms. Extensive research is currently focused on exploring prognostic biomarkers with the potential to improve clinical outcomes for GBM patients.

The extracellular matrix (ECM) is a vital structure that exists widely in all tissues, not only providing physical support (integrity and elasticity) but also maintaining tissue homeostasis by continuously remodeling dynamically [9]. In the tumor microenvironment (TME), the ECM is also an essential non–cellular component (CC) that can form a scaffold in tumor tissues and promote tumor progression and metastasis by interacting with adjacent cells, initiating various cell signaling cascades, and so on [10]. The ECM mainly consists of specialized proteins and connective tissue secreted by fibroblasts, forming a complex macromolecular network with unique physical, biochemical, and biological characteristics [11]. Numerous studies have shown that collagen, hyaluronic acid, laminin, and fibronectin in the ECM play a crucial role in tumor development [12–14]. In the initial stage of GBM, local ECM changes occur and gradually spread to the entire brain, and these changes further promote tumor infiltration in the later stage of GBM [15]. Studies have shown that GBM cells can create a more favorable environment for migration and invasion by adhering to and degrading the ECM via cell surface receptors [16]. GBM cells can synthesize and deposit matrix proteins, such as myosin-C, laminin, fibronectin, and Type IV collagen, to promote tumor cell movement [17]. Additionally, GBM produces a large amount of ECM metalloproteinase inducers, which can recruit metalloproteinases produced by matrix cells and activate gelatinase A, considered one of the critical factors in GBM progression [18]. Gulaia et al. found that the typical ECM component myosin-C expression level directly correlated with GBM tumor grading and patient prognosis [19]. Components related to cancer cell migration, such as the NG2 proteoglycan and syndecan-2, are also highly expressed in brain tumors, including GBM [20]. Overall, as an essential component of the TME that significantly affects the occurrence, development, and metastasis of GBM, the molecular mechanisms by which the ECM exerts its effects have not been fully elucidated, and the application of its related genes as prognostic tools for GBM has excellent potential and requires further research.

This study demonstrated the significant role of the ECM in GBM. Using bioinformatics methods based on ECM-related genes, we identified five prognostic biomarkers of GBM and established a risk model based on them. Further analysis included immune infiltration and drug prediction. This research is aimed at providing insight and support for subsequent GBM prognosis prediction and pathogenesis exploration studies.

## 2. Materials and Methods

### 2.1. Data Source

We acquired GBM-related RNA sequencing data from TCGA-GBM cohort available at the UCSC Xena database (http://xena.ucsc.edu/) as our training set. This dataset consisted of 154 GBM samples with survival information. For validation purposes, we utilized the mRNAseq325 dataset from the CGGA database (http://www.cgga.org.cn/), which included 85 GBM samples with comprehensive survival information. To identify genes associated with the ECM, we retrieved 1027 genes from different categories in the MSigDB database (https://www.gsea-msigdb.org/gsea/msigdb/).

### 2.2. ECM Index Calculation in TCGA-GBM Cohort

We used the “GSVA” software for single-sample gene set enrichment analysis (ssGSEA) to calculate GBM tumor ECM index. Subsequently, we classified GBM tumor samples into high- and low-ECM index groups based on the median ECM index.

### 2.3. Differentially Expressed Gene (DEG) Identification and Weighted Gene Coexpression Network Analysis (WGCNA)

Differential expression analysis was performed between the high- and low-ECM index groups in TCGA-GBM dataset using the “DESeq2” package software [21], with |log2FC| > 1 and adj.*p* < 0.05 as the filtering criteria. We then used the “WGCNA” package [22] to perform hierarchical clustering on the samples to detect and eliminate outliers. Next, we used the pickSoftThreshold function to select an appropriate soft threshold (*β*). We calculated Pearson correlations to identify the critical module most closely related to the ECM index. To screen out core genes, we applied the screening conditions of module membership (MM) > 0.7 and gene significance (GS) > 0.2 in the intramodule analysis.

### 2.4. Screening of Candidate Genes

We performed an intersection analysis between the DEGs and the core genes obtained from WGCNA to identify promising genes for further investigation. The resulting candidate genes were utilized to construct a protein–protein interaction (PPI) network using the STRING database (http://string-db.org) [23]. We conducted Gene Ontology (GO) and Kyoto Encyclopedia of Genes and Genomes (KEGG) pathway analyses to gain insights into the potential mechanisms associated with these candidate genes. These analyses were performed using the “clusterProfiler” package [24], with a significance threshold set at adj.*p* < 0.05.

### 2.5. Prognostic Gene Signature Establishment

We conducted univariate Cox analysis to identify genes associated with survival, using hazard ratio (HR) ≠ 1 and *p* < 0.05 as the screening criteria. Subsequently, we utilized the least absolute shrinkage and selection operator (LASSO) algorithm implemented through the “glmnet” package [25] to construct a risk model. The samples were categorized into high- and low-risk groups based on the risk scores. To evaluate the prognostic value of the risk model, Kaplan–Meier (KM) survival curve analysis was performed using the “survminer” R package [26]. The validation set underwent a similar process to that of the training set.

### 2.6. Cell-Experimental In Vitro Validation

#### 2.6.1. Cell Culture and Transfection

The U251 cells were grown in Dulbecco's modified Eagle medium (DMEM) supplemented with 10% fetal bovine serum (Gibco, United States) and 1% penicillin and streptomycin (P/S) (Gibco, United States). To examine the impact of these five genes on the invasiveness of the U251 cell line in terms of proliferation capacity, we created lentiviral vectors specific to each gene for transfection into the U251 cell line. The recombinant plasmids were created by introducing the *PLAUR*, bradykinin receptor B2 (*BDKRB2*), *ADAMTS14*, *FOSL1*, and tissue inhibitor of metalloproteinase 1 (*TIMP1*) genes into lentiviral vectors, with empty plasmids serving as controls. The target and packaging plasmids (psPAX2 and pMD2.G) were transfected into 293T cells using Lipo-2000 (Thermo Fisher, United States), and the viral supernatants were harvested and concentrated at 48 and 72 h posttransfection, respectively. The effectiveness of the transfection was assessed using reverse transcription quantitative polymerase chain reaction (RT-qPCR). The isolation of total RNA was performed using Trizol reagent (AG21101, China), and cDNA synthesis was carried out using a commercial reverse transcription kit (AG1706, China). The PCR reaction kit (AG11718, China) was used to evaluate the relative gene expression levels. The primer sequences mentioned are included in Table 1.

#### 2.6.2. The CCK-8 Test Measures Cell Proliferation

The cells are cultivated to a certain concentration of cell suspension, with each well containing 2 × 10^3^ cells in an optimal condition of growth. The culture base was supplemented with 100 *μ*L, and 96 porous cell cultivation boards were added. The cultivation was carried out in a 37°C cultivation box containing 5% carbon dioxide. At 0, 24, and 48 h, CCK-8 solution was added and incubated for 30 min in the cultivation boxes. The absorption at 450 nm was measured by enzyme detection.

#### 2.6.3. Transwell Assay for Cell Invasiveness

The Matrigel adhesive is evenly applied to the upper surface of the lower membrane of the Transwell chamber. It is then incubated in a cultivation box at a temperature of 37°C for a duration of 3 h. This allows the basal polymer to form a thin film and absorb any excess fluid. Next, 100 *μ*L of serum-free cultivation containing 1 × 10^4^/mL is added to the top layer of the chamber, while the lower chamber is filled with 500 *μ*L of complete cultivation containing serum. The cells are then placed in a cell culture box for a period of 72 h. During this time, the cells exhibit a crystalline purple color and can be observed under a microscope using the Transwell cell chamber.

#### 2.6.4. Wound Healing Assay

The cells were injected in 6-well culture plates at roughly 5 × 10^5^ cells per well, achieving a cell density growth value of 80%, and were assessed using a 200-*μ*L tip positioned perpendicularly to a horizontal line on the reverse side of the six-well plate. Cells were rinsed with phosphate-buffered saline (PBS) to eliminate unattached cells, low-serum (2%) media were introduced, and the six-well plates were incubated at 37°C in a 5% CO_2_ environment. The cells were examined under a microscope at 0 and 24 h, respectively, and photographed.

### 2.7. Immune Analysis in Risk Groups

We utilized the “estimate” package [27] to assess the immune score, stromal score, ESTIMATE score, and tumor purity. For comparing the proportion of 22 infiltrated immune cells between the two groups, we employed the CIBERSORT algorithm [28] based on RNA sequencing (RNA-Seq) data. Furthermore, we analyzed the differential expression of immune checkpoints in the two groups.

### 2.8. Long noncoding RNA (lncRNA)–MicroRNA (miRNA)–Messenger RNA (mRNA) Network Construction

We initially conducted a screening process to identify differentially expressed lncRNAs (DElncRNAs) between the high- and low-ECM index groups. This analysis used the “DESeq2” package in R software, with the |log2FC| > 1 and adj criteria *p* < 0.05. Next, we utilized the miRNA database (https://www.mirnet.ca/) to predict interactions between miRNAs and both the mRNAs in the risk model and the DElncRNAs. By overlapping the miRNAs involved in these interactions, we identified target miRNAs. Lastly, we constructed the lncRNA–miRNA–mRNA network using Cytoscape.

### 2.9. Nomogram Construction and Evaluation

We used univariate Cox, proportional hazards assumption, and multivariate Cox analyses to identify independent prognostic factors (IPFs) and visualized the results using the “forest plot” package. We then performed receiver operating characteristic (ROC) analysis to test the IPFs and generated a nomogram using the “rms” package. Finally, we estimated the nomogram's performance using a calibration curve for 0.5-, 1.5-, and 2.5-year OS.

## 3. Results

### 3.1. DEG Identification and WGCNA

**Figure 1:**
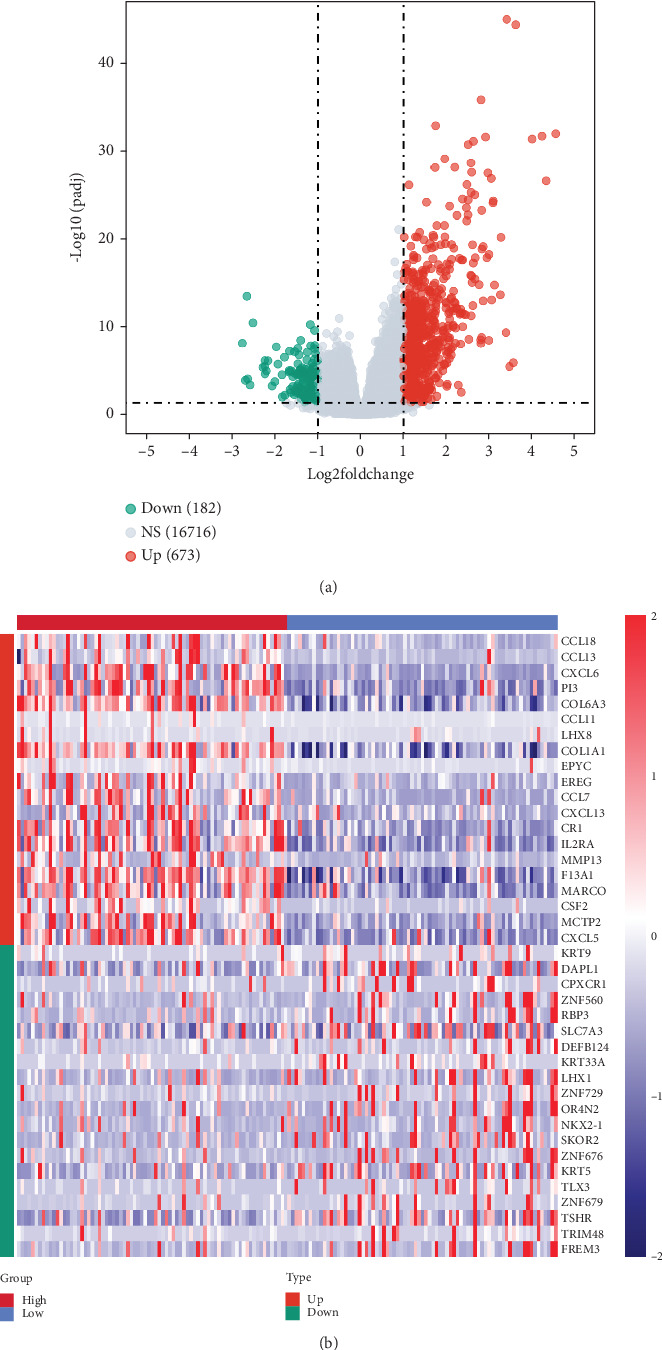
The high- and low-ECM score groups were screened for differential genes. (a) Volcanogram. (b) Heat map.

**Figure 2:**
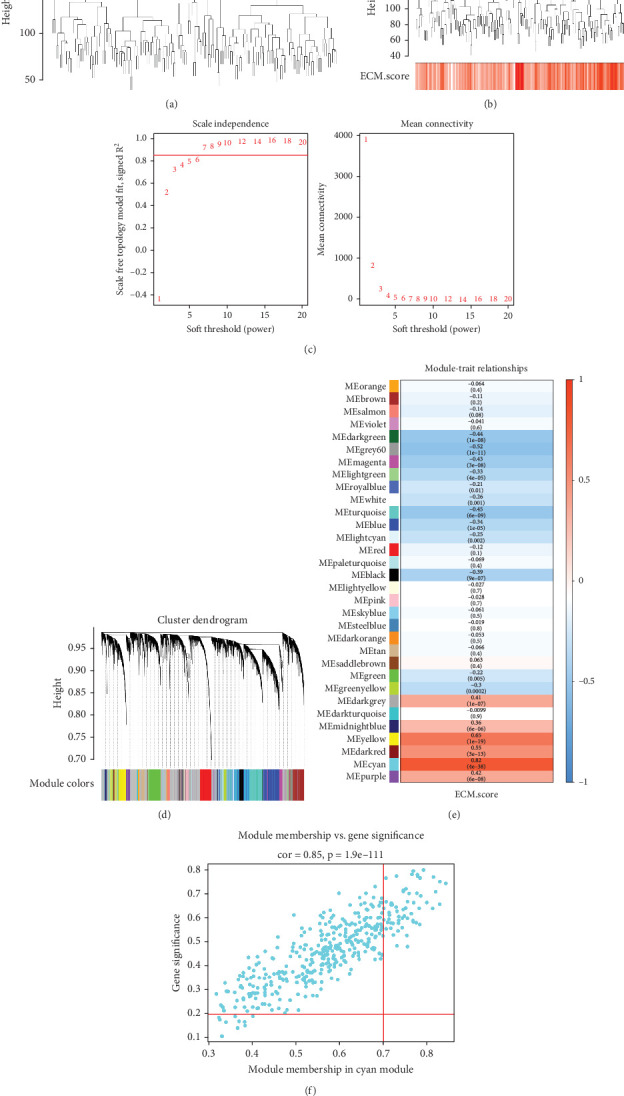
Construction of coexpression network by WGCNA. (a, b) Analysis of outlier samples. (c) Selection of soft threshold power (*β*). (d) Cluster dendrogram. (e) Module–trait relationship gene module showing the correlations between eigengene and phenotype. (f) The correlation between genes and coexpression modules (the horizontal axis) and between genes and phenotypes (the vertical axis).

We identified 855 DEGs between the high- and low-ECM index groups (Figure 1(a)) and examined the expression of the top 20 up- and downregulated genes (sorted by log_2_FC) (Figure 1(b)). We detected two outliers using the Euclidean distance method (Figures 2(a) and 2(b)). Using a soft threshold (*β*) of 7 (*R*^2^ = 0.85), we identified 32 modules (Figures 2(c) and 2(d)), with the cyan module showing the strongest correlation with the ECM index (|Cor| = 0.82, *p* = 4e − 38; Figure 2(e)). We screened out 59 core genes from the cyan module based on the screening conditions of gene significance and module membership (Figure 2(f)).

### 3.2. Forty-Five Candidate Genes Were Sifted

**Figure 3:**
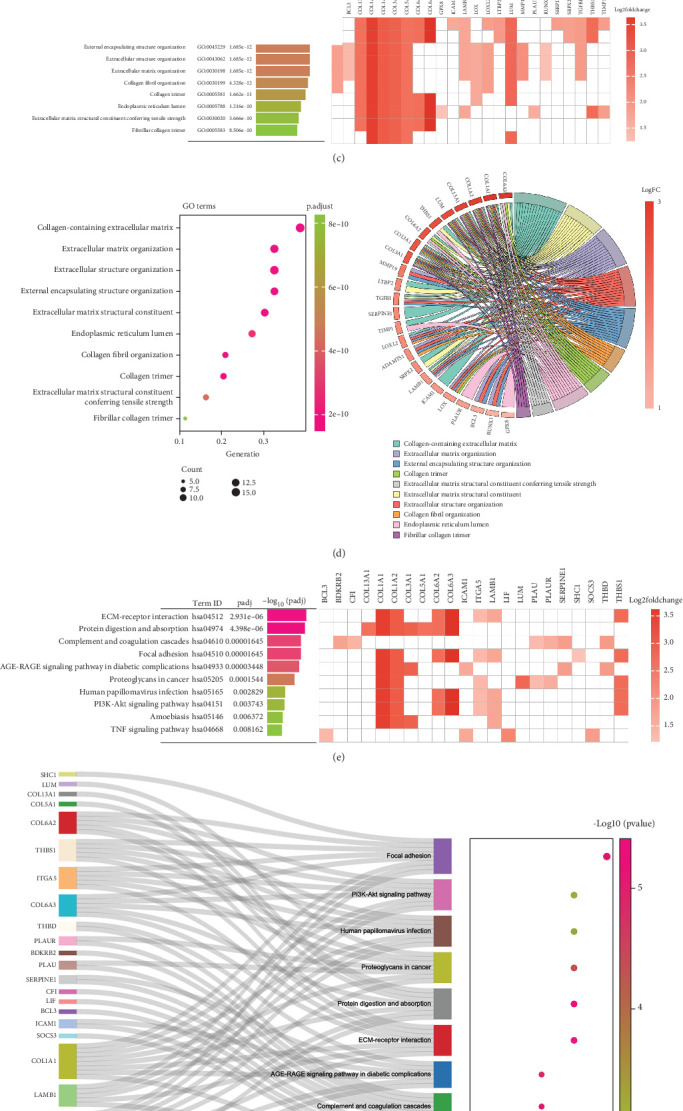
GO and KEGG analyses. (a) The Venn diagram. (b) The PPI network (c, d). GO terms. (e, f) EGG pathway.

We used 45 overlapping genes from the 59 WGCNA core genes and 855 DEGs as candidate genes (Figure 3(a)). The PPI network of the candidate genes is shown in Figure 3(b). In our study, the candidate genes were significantly enriched in 254 biological processes (BPs), 18 CCs, 27 molecular functions (MFs), and 11 KEGG pathways (Tables S1 and S2). The GO terms enriched included “endoplasmic reticulum lumen,” “fibrillar collagen trimer,” and “collagen-containing extracellular matrix” (Figures 3(c) and 3(d)). In contrast, the enriched KEGG pathways included “amoebiasis,” “complement and coagulation cascades,” and “focal adhesion” (Figures 3(e) and 3(f)).

### 3.3. Screening and Validation of Candidate Genes and Risk Prediction Model of GBM Based on the Candidate Genes

**Figure 4:**
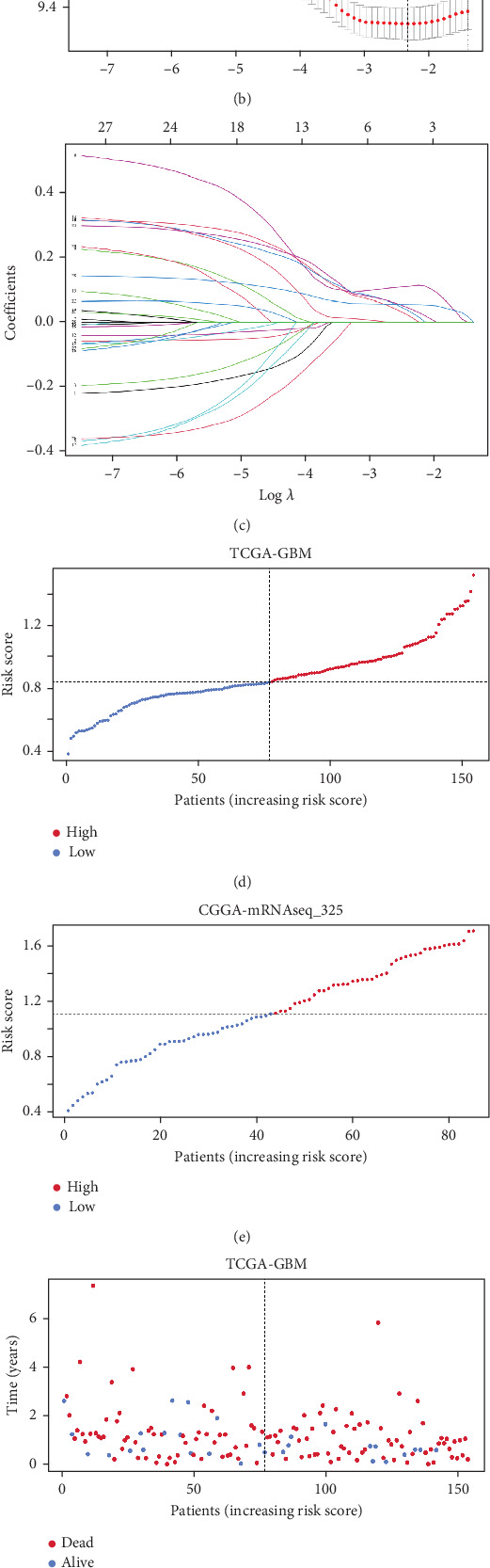
ECM-related characteristics. (a) Univariate Cox regression analysis. (b, c) LASSO regression analysis. (d–g) Distribution of risk scores and survival times with the risk scores. (h, i) Overall survival curves of TCGA and CGGA between the two groups.

We performed univariate Cox analysis to identify candidate genes with prognostic correlation and identified 28 genes with HR ≠ 1 and *p* < 0.05. We further filtered these genes using the LASSO algorithm (Figures 4(a), 4(b), and 4(c)). Based on these results, we constructed a five-gene risk model with the following formula: risk score = (0.111∗*PLAUR*) + (0.013∗*BDKRB*2) + (0.028∗*ADAMTS*14) + (0.039∗*FOSL*1) + (0.054∗*TIMP*1). We allocated patients into high- and low-risk groups (77 patients in each group) (Figures 4(d) and 4(f)). The results were consistent when GBM patients from the mRNAseq325 dataset were categorized into high-risk (*n* = 42) and low-risk (*n* = 43) groups (Figures 4(e) and 4(g)). We found that the Kaplan–Meier (KM) analysis clearly distinguished the prognosis between the two groups (Figures 4(h) and 4(i)).

### 3.4. Experimental Validation of Screening Genes

**Figure 5:**
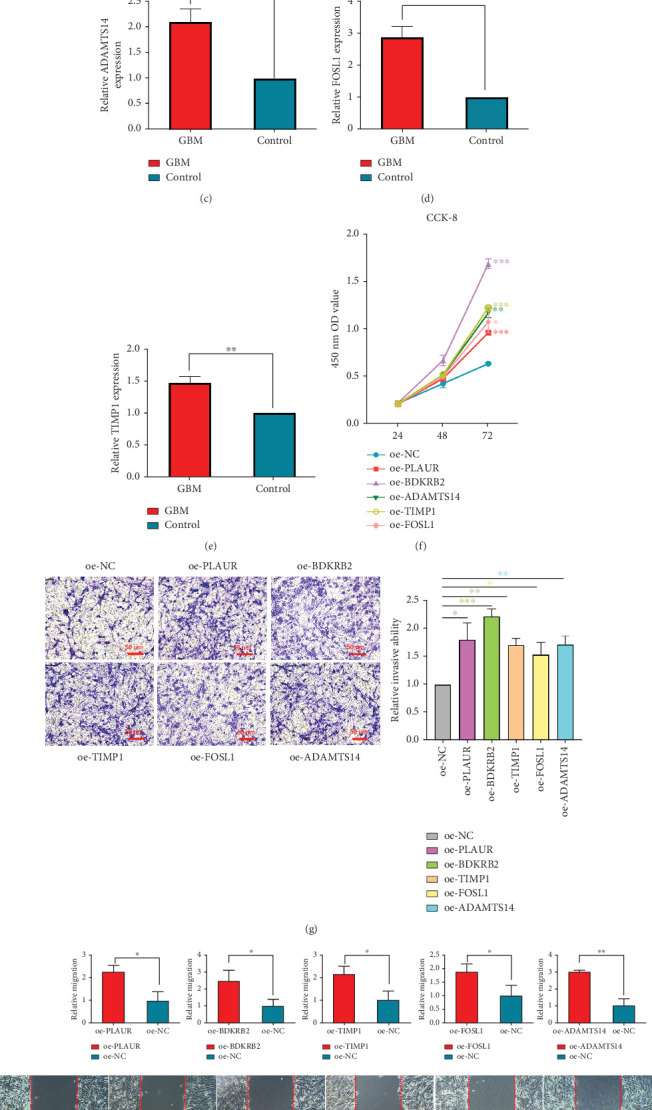
Validation of the effects of the screened genes on U251 cells. (a–e) RT-qPCR showed that *PLAUR*, *BDKRB2*, *ADAMTS14*, *FOSL1*, and *TIMP1* had high transfection efficiency in U251 cell line. (f) CCK-8 assay demonstrated that *PLAUR*, *BDKRB2*, *ADAMTS14*, *FOSL1*, and *TIMP1* enhanced the proliferation of U251. (g) Transwell assay indicated that *PLAUR*, *BDKRB2*, *ADAMTS14*, *FOSL1*, and *TIMP1* enhance the invasive ability of U251. ⁣^∗^*p* < 0.05, ⁣^∗∗^*p* < 0.01, and ⁣^∗∗∗^*p* < 0.001. (h) Wound healing assays showed that overexpression of *PLAUR*, *BDKRB2*, *ADAMTS14*, *FOSL1*, and *TIMP1* elevated the migration potential of U251 cells.

The RT-qPCR test demonstrated successful transfection of *PLAUR* (Figure 5(a)), *BDKRB2* (Figure 5(b)), *ADAMTS14* (Figure 5(c)), *FOSL1* (Figure 5(d)), and *TIMP1* (Figure 5(e)) into the U251 cell line. The Cell Counting Kit-8 (CCK-8) assay demonstrated that overexpression of *PLAUR*, *BDKRB2*, *ADAMTS14*, *FOSL1*, and *TIMP1* increased the proliferative capacity of U251 cells (Figure 5(f)). The Transwell assay indicated that overexpression of *PLAUR*, *BDKRB2*, *ADAMTS14*, *FOSL1*, and *TIMP1* boosted the invasive potential of U251 cells (Figure 5(g)). Wound healing assays showed that overexpression of *PLAUR*, *BDKRB2*, *ADAMTS14*, *FOSL1*, and *TIMP1* elevated the migration potential of U251 cells (Figure 5(h)).

### 3.5. Six Distinct Immune Cells Were Discovered

**Figure 6:**
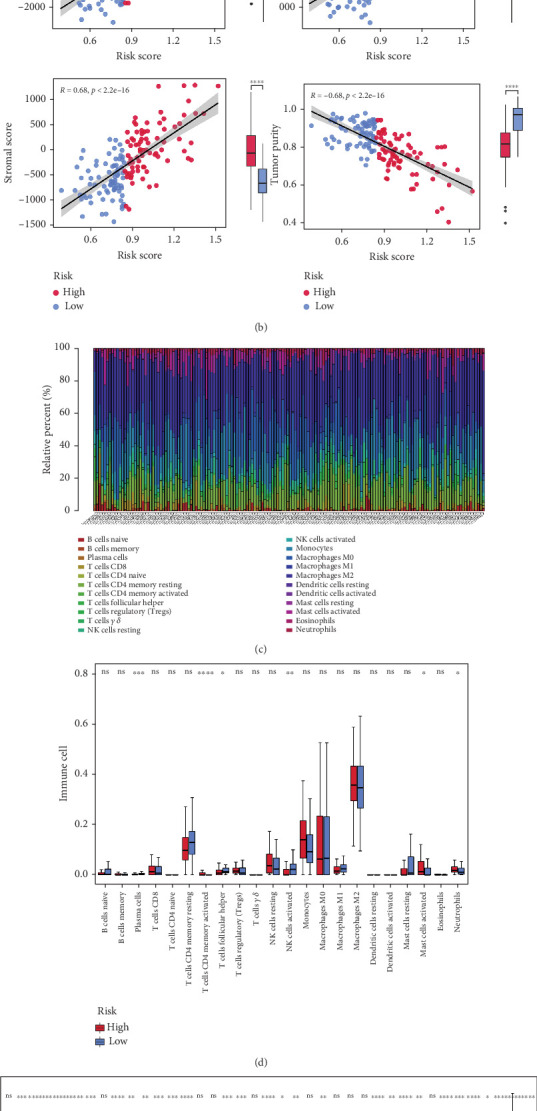
Analysis of the tumor-immune microenvironment. (a) Systematic estimation of risk models and clinical factors. (b) correlation analysis of risk score with ESTIMATE score, immune score, stromal score, and tumor purity. (c) Proportion of immune cells. (d) Differences in the distribution of tumor-infiltrating immune cells. (e) Differences in immune checkpoints.

We discovered a significant difference in risk score across subgroups defined by age and tumor status, but not by gender or radiation (Figure 6(a)). Furthermore, there was a strong correlation between the immunological, stromal, and estimation scores and the risk score, with notably elevated levels observed in the high-risk group (Figure 6(b)). Furthermore, we detected significant differences in six cell types (helper follicular T cells, activated memory CD4 T cells, activated mast cells, activated natural killer (NK) cells, plasma cells, and neutrophils) between the risk groups (Figures 6(c) and 6(d)). We also found that 29 immune checkpoints showed remarkable differences between the groups (Figure 6(e)).

### 3.6. Construction of lncRNA–miRNA–mRNA Network

**Figure 7:**
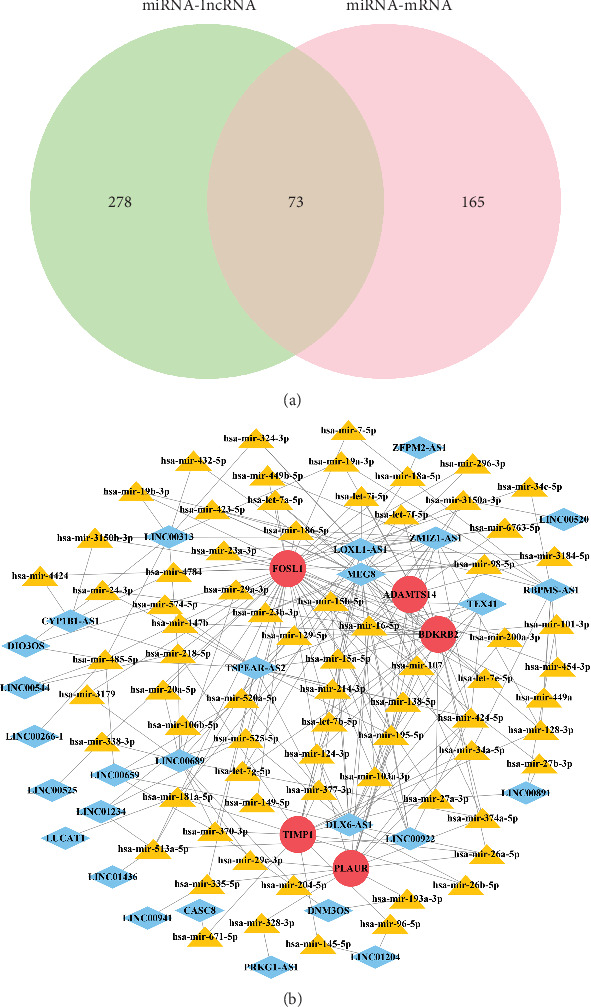
(a) Analysis of target miRNAs. (b) The ceRNA regulatory network.

We identified 547 DElncRNAs between the high- and low-ECM index groups. Using the miRNet database, we predicted 351 miRNAs interacting with the DElncRNAs and 238 miRNAs interacting with the model mRNAs (Tables S3 and S4). We intersected the 351 miRNAs of DElncRNAs with the 238 miRNAs of model mRNAs to obtain 73 target miRNAs (Figure 7(a)). We then constructed a competing endogenous RNA (ceRNA) network of 27 lncRNAs, 73 miRNAs, and 5 model mRNAs (Figure 7(b)).

### 3.7. Construction and Evaluation of the Predictive Nomogram

**Figure 8:**
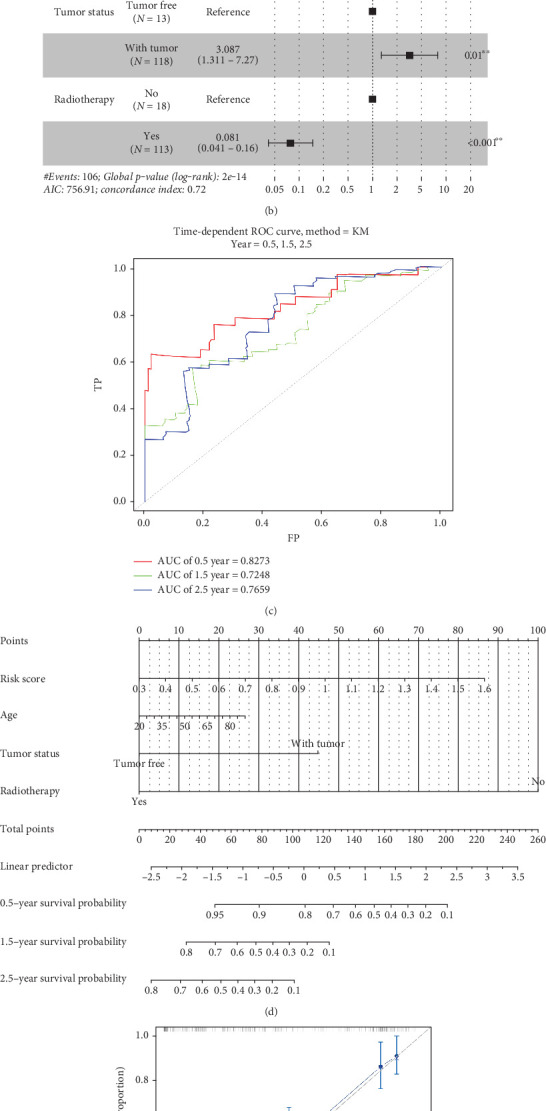
Evaluation of prognostic signature to predict the OS of GBM patients. (a) Univariate Cox regression analysis. (b) Multivariate Cox regression analysis. (c) ROC curve of the 0.5-, 1.5-, and 2.5-year OS for GBM patients. (d) Nomogram for predicting 0.5-, 1.5-, and 2.5-year OS for GBM patients. (e) The calibration plots of the nomogram predicting 0.5-year, 1.5-year, and 3.5-year OS.

Finally, we generated a prognostic model based on four IPFs: risk score, age, tumor status, and radiotherapy (Figures 8(a) and 8(b)). The area under the curve (AUC) values for 0.5-, 1.5-, and 2.5-year OS were 0.8273, 0.7248, and 0.7659, respectively (Figure 8(c)). We constructed a nomogram for 0.5-, 1.5-, and 2.5-year OS and confirmed its performance using a calibration curve (Figures 8(d) and 8(e)).

## 4. Discussion

GBM is one of the common malignant brain tumors in adults. Although surgery and chemotherapy can improve the prognosis of patients to some extent, the overall prognosis of GBM remains extremely poor [29]. Several studies have shown a direct correlation between changes in the brain ECM and rapid progression of GBM [30, 31]. As an essential component of the TME, the ECM causes changes in the TME and gradually spreads to the entire brain tissue. These changes are related to the interaction between the ECM and GBM cells, which biologically alter the properties of GBM cells [32]. In this study, we used bioinformatics methods based on ECM-related genes to identify prognostic genes of GBM and established a predictive model based on them. We further conducted a series of analyses to explore the role of ECM-related genes in GBM prognosis.

We found that 45 candidate genes highly correlated with ECM and GBM significantly enriched in 299 GO entries and 11 KEGG pathways through functional enrichment analysis based on GO and KEGG databases. Apart from entries related to ECM structure and organization, these genes significantly enriched collagen fibril organization, collagen trimer, and endoplasmic reticulum lumen in the GO results. Collagen protein is the primary component of ECM, and in gliomas, mainly Type I and IV collagen is produced, which provides a scaffold for glioma cell migration and alters the mechanical strength of the tumor tissue, thus enhancing the invasive ability of tumor cells [33, 34]. Studies have shown that glioma cells migrate farther in high-concentration collagen gel than in low-concentration collagen gel, which confirms the significant role of collagen in promoting tumor cell migration [35]. Endoplasmic reticulum stress can activate autophagy, and when endoplasmic reticulum stress lasts too long or is too intense, autophagy is overactivated, leading ultimately to cell apoptosis, as demonstrated in many studies on gliomas [36, 37]. In addition, candidate genes' main KEGG enrichment results are significantly associated with the complement system, PI3K-AKT signaling pathway, proteoglycans, TNF signaling pathway, and others. The complement system is a critical component of the innate immune system, consisting of over 30 proteins, among which complement C3a can trigger the activation and migration of inflammatory cells [38]. Overactivation of C3a can cause various diseases and tumors, including ovarian cancer, lung cancer, melanoma, and medulloblastoma [39–41]. The phosphatidylinositol 3-kinase (PI3K)–mediated signaling pathway is one of the three major signaling transduction pathways involved in GBM [42]. PI3K regulates cancer cell inflammation, survival, metabolism, migration, invasion, and development. Meanwhile, it can inhibit the activity of various proapoptotic proteins by inducing the activation of Akt. It can then suppress cell apoptosis by inhibiting the release of cytochrome C and the movement caspase-3, thereby promoting tumor survival and progression [43, 44]. Proteoglycans regulate the activity of many signaling pathways, including PI3K-AKT and PDGFR-*α*, and can promote the interaction between tumors and their microenvironment [45, 46]. At the same time, studies have shown that proteoglycans and related enzymes (such as CSPG4/NG2, VCAN, and HS) are upregulated in GBM and significantly affect its occurrence and development [47–49]. Tumor necrosis factor (TNF) and TNF receptor superfamily (TNFRSF) play crucial roles in GBM, and many studies have shown that TNF-*α*, TNFRSF19, etc. are upregulated in GBM and significantly promote GBM cell invasion [50, 51].

To further identify critical genes affecting the prognosis of GBM, we used single-factor Cox regression analysis and the LASSO algorithm to screen for five prognostic genes (*PLAUR*, *BDKRB2*, *ADAMTS14*, *FOSL1*, and *TIMP1*) and constructed a risk model. *PLAUR* is the receptor of plasminogen activator (PLAU), which participates in various physiological functions such as wound healing and is also associated with rheumatoid arthritis, Quebec platelet disorder, and tumor angiogenesis and metastasis [52–54]. Multiple studies have shown that PLAU and *PLAUR* are highly expressed in gliomas and promote glioma cell invasion, tumor growth, and angiogenesis [55]. *BDKRB2* can promote tumor progression through multiple pathways, and its expression level is often positively correlated with the malignant characteristics of tumor [56]. *BDKRB2* can promote angiogenesis by increasing vascular permeability and upregulating vascular endothelial growth factor (VEGF) [57] and can also promote tumor cell migration and invasion through TRPM7, MMP2, endothelin-1, and ERK signaling pathways [58]. According to Yang et al. [59], *BDKRB2* is substantially related to epithelial–mesenchymal transition (EMT) and enhances the occurrence and progression of gliomas. A disintegrin and metalloproteinase with thrombospondin motifs (ADAMTS), a member of the extracellular zinc metalloproteinase family, is highly associated with arthritis, atherosclerosis, reproduction, and cancer [60]. Studies have shown that *ADAMTSL4* is associated with immune-related processes in GBM and reflects the infiltration of complex immune cells and TME environment in tumor [61]. At the same time, *ADAMTS14* is significantly upregulated in breast cancer and can lead to the progression of oral and liver cell carcinomas [62]. Chen et al. found that *ADAMTS14* is carcinogenic in clear cell renal cell carcinoma and is significantly associated with immunity [63]. *FOSL1* is an AP-1 transcription factor that has prognostic value in human solid tumors such as breast cancer, lung cancer, pancreatic cancer, and colon cancer, and its overexpression is associated with tumor progression or reduced patient survival rates [64, 65]. Multiple studies have shown that *FOSL1* is responsible for maintaining the growth of in vitro glioma cells and promoting glioma cell invasion. It is an adverse prognostic factor for GBM patients and can promote UBC9-dependent CYLD SUMOylation, inducing proneural–mesenchymal transition (PMT) in GBM stem cells [66]. *TIMP1* is highly expressed in most cancers and functions in a soluble form. In tumors such as gastric, pancreatic, colorectal, breast, melanoma, and thyroid cancer, *TIMP1* is often significantly associated with poor prognosis [67]. In gliomas, overexpression of *TIMP1* reduces the effectiveness of topoisomerase inhibitors in GBM. It may also reduce the growth of spheroids, which could affect the efficacy of the topoisomerase one inhibitor irinotecan [68]. In summary, *PLAUR*, *BDKRB2*, *ADAMTS14*, *FOSL1*, and *TIMP1* have all been shown to be involved in the occurrence and development of GBM or related diseases and have the potential to be further developed as prognostic biomarkers.

The ECM is an essential component of the TME, and immune cells within the TME are another significant component that affects the progression of GBM. This study discovered six immune cells with horizontal differences between high- and low-risk groups using CIBERSORT. Activated CD4 memory T cells were found to be significantly positively correlated with biomarkers, while activated NK cells were negatively associated with biomarkers. Most cells infiltrating the tumor are T cells (accounting for 80%), and there is a correlation between the degree of lymphocyte infiltration in the primary tumor and metastasis [69]. T cells mainly include CD8 and CD4 T cells, which can receive antigen presentation from dendritic cells, inducing tumor-specific responses [70]. There is ample evidence of glioma-induced immune suppression. Authier et al.'s treatment-related immune suppression study shows that treated glioma cells are more immune-suppressive and form tumors faster in in vitro and animal models [71]. Furthermore, research has found that activated CD4 memory T cells are enhanced in the brains of GBM-bearing mice, suggesting their involvement in immune processes in GBM [72]. GBM immune escape is fueled by immunosuppressive hypoxic TME, frequently unfavorable to NK cell activity. The action of ecto-5⁣′-nucleotidase (CD73), a hypoxia ectoenzyme, is partly responsible for the immunological, metabolic imbalance of NK cell function in GBM. Wang et al. discovered that CD73 is related to GBM prognosis and can induce extracellular adenosine (ADO) buildup, resulting in purinergic signaling-mediated NK cell activity damage [73, 74].

## 5. Conclusions

This study identified five ECM-related characteristic genes and established a risk model that accurately predicts GBM prognosis. Additionally, this study revealed the related mechanism of ECM regulation of pleomorphic GBM. However, our conclusions are limited to bioinformatics and require further validation and exploration through in vitro and in vivo experiments. Therefore, we plan to conduct further investigation and validation of the conclusions of this study by designing in vitro molecular experiments and gene knockout animal models to provide a solid foundation and basis for the clinical treatment and pathogenesis exploration of GBM.

## Figures and Tables

**Table 1:** Primers for RT-qPCR.

**Gene**	**Forward primer (5**⁣′**-3**⁣′**)**	**Reverse primer (5**⁣′**-3**⁣′**)**
*PLAUR*	TGTAAGACCAACGGGGATTGC	AGCCAGTCCGATAGCTCAGG
*BDKRB2*	GTCTGTTCGTGAGGACTCCG	AAAGGTCCCGTTAAGAGTGGG
*ADAMTS14*	CCAATCGGAGGTTGGTAGTGC	CACACACTCCTGCCGTAAGG
*FOSL1*	CAGGCGGAGACTGACAAACTG	TCCTTCCGGGATTTTGCAGAT
*TIMP1*	AGAGTGTCTGCGGATACTTCC	CCAACAGTGTAGGTCTTGGTG

## Data Availability

The datasets used in this study can be accessed from TCGA count (https://portal.gdc.cancer.gov) and the CGGA database (http://www.cgga.org.cn/).

## Supporting Information

Supporting Information 1 Table S1: GO analysis of differential genes.

Supporting Information 2 Table S2: KEGG analysis of differential genes.

Supporting Information 3 Table S3: Network of lncRNA–mRNA relationships.

Supporting Information 4 Table S4: Network of miRNA–mRNA relationships.

## References

[1] Davis M. E. (2018). Epidemiology and overview of gliomas. *Seminars in Oncology Nursing*.

[2] Tykocki T., Eltayeb M. (2018). Ten-year survival in glioblastoma. A systematic review. *Journal of Clinical Neuroscience*.

[3] Hanif F., Muzaffar K., Perveen K., Malhi S. M., Simjee ShU (2017). Glioblastoma multiforme: a review of its epidemiology and pathogenesis through clinical presentation and treatment. *Asian Pacific Journal of Cancer Prevention: APJCP*.

[4] Louis D. N., Perry A., Wesseling P. (2021). The 2021 WHO classification of tumors of the central nervous system: a summary. *Neuro-Oncology*.

[5] Huang B., Li X., Li Y., Zhang J., Zong Z., Zhang H. (2021). Current immunotherapies for glioblastoma multiforme. *Frontiers in Immunology*.

[6] Baer J. C., Freeman A. A., Newlands E. S., Watson A. J., Rafferty J. A., Margison G. P. (1993). Depletion of O^6^-alkylguanine-DNA alkyltransferase correlates with potentiation of temozolomide and CCNU toxicity in human tumour cells. *British Journal of Cancer*.

[7] Grossmann P., Narayan V., Chang K. (2017). Quantitative imaging biomarkers for risk stratification of patients with recurrent glioblastoma treated with bevacizumab. *Neuro-Oncology*.

[8] Wu W., Klockow J. L., Zhang M. (2021). Glioblastoma multiforme (GBM): an overview of current therapies and mechanisms of resistance. *Pharmacological Research*.

[9] Theocharis A. D., Skandalis S. S., Gialeli C., Karamanos N. K. (2016). Extracellular matrix structure. *Advanced Drug Delivery Reviews*.

[10] Yamaguchi R., Perkins G. (2018). Animal models for studying tumor microenvironment (TME) and resistance to lymphocytic infiltration. *Cancer Biology & Therapy*.

[11] Bourboulia D., Stetler-Stevenson W. G. (2010). Matrix metalloproteinases (MMPs) and tissue inhibitors of metalloproteinases (TIMPs): positive and negative regulators in tumor cell adhesion. *Seminars in Cancer Biology*.

[12] Karalis T., Skandalis S. S. (2022). Hyaluronan network: a driving force in cancer progression. *American Journal of Physiology. Cell Physiology*.

[13] Martins Cavaco A. C., Dâmaso S., Casimiro S., Costa L. (2020). Collagen biology making inroads into prognosis and treatment of cancer progression and metastasis. *Cancer Metastasis Reviews*.

[14] Rick J. W., Chandra A., Dalle Ore C., Nguyen A. T., Yagnik G., Aghi M. K. (2019). Fibronectin in malignancy: cancer-specific alterations, protumoral effects, and therapeutic implications. *Seminars in Oncology*.

[15] Restall I., Bozek D. A., Chesnelong C., Weiss S., Luchman H. A. (2018). Live-cell imaging assays to study glioblastoma brain tumor stem cell migration and invasion. *Journal of Visualized Experiments: JoVE*.

[16] Venkataramani V., Yang Y., Schubert M. C. (2022). Glioblastoma hijacks neuronal mechanisms for brain invasion. *Cell*.

[17] Erices J. I., Bizama C., Niechi I. (2023). Glioblastoma microenvironment and invasiveness: new insights and therapeutic targets. *International Journal of Molecular Sciences*.

[18] Yu-Ju Wu C., Chen C. H., Lin C. Y. (2020). CCL5 of glioma-associated microglia/macrophages regulates glioma migration and invasion via calcium-dependent matrix metalloproteinase 2. *Neuro-Oncology*.

[19] Gulaia V., Kumeiko V., Shved N. (2018). Molecular mechanisms governing the stem cell’s fate in brain cancer: factors of stemness and quiescence. *Frontiers in Cellular Neuroscience*.

[20] Trotter J., Karram K., Nishiyama A. (2010). NG2 cells: properties, progeny and origin. *Brain Research Reviews*.

[21] Love M. I., Huber W., Anders S. (2014). Moderated estimation of fold change and dispersion for RNA-seq data with DESeq2. *Genome Biology*.

[22] Langfelder P., Horvath S. (2008). WGCNA: an R package for weighted correlation network analysis. *BMC Bioinformatics*.

[23] von Mering C., Huynen M., Jaeggi D., Schmidt S., Bork P., Snel B. (2003). STRING: a database of predicted functional associations between proteins. *Nucleic Acids Research*.

[24] Yu G., Wang L. G., Han Y., He Q. Y. (2012). clusterProfiler: an R package for comparing biological themes among gene clusters. *OMICS: A Journal of Integrative Biology*.

[25] Zhang M., Zhu K., Pu H. (2019). An immune-related signature predicts survival in patients with lung adenocarcinoma. *Frontiers in Oncology*.

[26] Dong B., Liang J., Li D. (2021). Identification of a prognostic signature associated with the homeobox gene family for bladder cancer. *Frontiers in Molecular Biosciences*.

[27] Yoshihara K., Shahmoradgoli M., Martínez E. (2013). Inferring tumour purity and stromal and immune cell admixture from expression data. *Nature Communications*.

[28] Newman A. M., Liu C. L., Green M. R. (2015). Robust enumeration of cell subsets from tissue expression profiles. *Nature Methods*.

[29] Herrlinger U., Tzaridis T., Mack F. (2019). Lomustine-temozolomide combination therapy versus standard temozolomide therapy in patients with newly diagnosed glioblastoma with methylated MGMT promoter (CeTeG/NOA-09): a randomised, open-label, phase 3 trial. *The Lancet*.

[30] Bastiancich C., Danhier P., Préat V., Danhier F. (2016). Anticancer drug-loaded hydrogels as drug delivery systems for the local treatment of glioblastoma. *Journal of Controlled Release: Official Journal of the Controlled Release Society*.

[31] Virga J., Szivos L., Hortobágyi T. (2019). Extracellular matrix differences in glioblastoma patients with different prognoses. *Oncology Letters*.

[32] Mahesparan R., Read T. A., Lund-Johansen M., Skaftnesmo K., Bjerkvig R., Engebraaten O. (2003). Expression of extracellular matrix components in a highly infiltrative in vivo glioma model. *Acta Neuropathologica*.

[33] Belousov A., Titov S., Shved N. (2019). The extracellular matrix and biocompatible materials in glioblastoma treatment. *Frontiers in Bioengineering and Biotechnology*.

[34] Kaufman L. J., Brangwynne C. P., Kasza K. E. (2005). Glioma expansion in collagen I matrices: analyzing collagen concentration-dependent growth and motility patterns. *Biophysical Journal*.

[35] Bartoszewska S., Collawn J. F., Bartoszewski R. (2022). The role of the hypoxia-related unfolded protein response (UPR) in the tumor microenvironment. *Cancers*.

[36] Chang C. Y., Li J. R., Wu C. C. (2020). Endoplasmic reticulum stress contributes to indomethacin-induced glioma apoptosis. *International Journal of Molecular Sciences*.

[37] Peñaranda-Fajardo N. M., Meijer C., Liang Y. (2019). ER stress and UPR activation in glioblastoma: identification of a noncanonical PERK mechanism regulating GBM stem cells through SOX2 modulation. *Cell Death & Disease*.

[38] Li K., Anderson K. J., Peng Q. (2008). Cyclic AMP plays a critical role in C3a-receptor-mediated regulation of dendritic cells in antigen uptake and T-cell stimulation. *Blood*.

[39] Cho M. S., Vasquez H. G., Rupaimoole R. (2014). Autocrine effects of tumor-derived complement. *Cell Reports*.

[40] Gong B., Guo D., Zheng C. (2022). Complement C3a activates astrocytes to promote medulloblastoma progression through TNF-*α*. *Journal of Neuroinflammation*.

[41] Nabizadeh J. A., Manthey H. D., Steyn F. J. (2016). The complement C3a receptor contributes to melanoma tumorigenesis by inhibiting neutrophil and CD4+ T cell responses. *Journal of Immunology*.

[42] Luo S. M., Tsai W. C., Tsai C. K., Chen Y., Hueng D. Y. (2021). ARID4B knockdown suppresses PI3K/AKT signaling and induces apoptosis in human glioma cells. *Oncotargets and Therapy*.

[43] Chekenya M., Krakstad C., Svendsen A. (2008). The progenitor cell marker NG2/MPG promotes chemoresistance by activation of integrin-dependent PI3K/Akt signaling. *Oncogene*.

[44] Huang K. H., Fang W. L., Li A. F. Y. (2018). Caspase-3, a key apoptotic protein, as a prognostic marker in gastric cancer after curative surgery. *International Journal of Surgery (London, England)*.

[45] Phillips J. J., Huillard E., Robinson A. E. (2012). Heparan sulfate sulfatase SULF2 regulates PDGFR*α* signaling and growth in human and mouse malignant glioma. *The Journal of Clinical Investigation*.

[46] Svendsen A., Verhoeff J. J. C., Immervoll H. (2011). Expression of the progenitor marker NG2/CSPG4 predicts poor survival and resistance to ionising radiation in glioblastoma. *Acta Neuropathologica*.

[47] Asher R. A., Morgenstern D. A., Shearer M. C., Adcock K. H., Pesheva P., Fawcett J. W. (2002). Versican is upregulated in CNS injury and is a product of oligodendrocyte lineage cells. *The Journal of Neuroscience: The Official Journal of the Society for Neuroscience*.

[48] Badr C. E., Wurdinger T., Nilsson J. (2011). Lanatoside C sensitizes glioblastoma cells to tumor necrosis factor-related apoptosis-inducing ligand and induces an alternative cell death pathway. *Neuro-Oncology*.

[49] Su G., Meyer K., Nandini C. D., Qiao D., Salamat S., Friedl A. (2006). Glypican-1 is frequently overexpressed in human gliomas and enhances FGF-2 signaling in glioma cells. *The American Journal of Pathology*.

[50] Andreasen P. A., Egelund R., Petersen H. H. (2000). The plasminogen activation system in tumor growth, invasion, and metastasis. *Cellular and Molecular Life Sciences: CMLS*.

[51] Paulino V. M., Yang Z., Kloss J. (2010). TROY (TNFRSF19) is overexpressed in advanced glial tumors and promotes glioblastoma cell invasion via Pyk2-Rac1 signaling. *Molecular Cancer Research*.

[52] Dinesh P., Rasool M. (2018). uPA/uPAR signaling in rheumatoid arthritis: shedding light on its mechanism of action. *Pharmacological Research*.

[53] Gondi C. S., Lakka S. S., Yanamandra N. (2003). Expression of antisense uPAR and antisense uPA from a bicistronic adenoviral construct inhibits glioma cell invasion, tumor growth, and angiogenesis. *Oncogene*.

[54] Su S. C., Lin C. W., Yang W. E., Fan W. L., Yang S. F. (2016). The urokinase-type plasminogen activator (uPA) system as a biomarker and therapeutic target in human malignancies. *Expert Opinion on Therapeutic Targets*.

[55] Raghu H., Nalla A. K., Gondi C. S., Gujrati M., Dinh D. H., Rao J. S. (2012). uPA and uPAR shRNA inhibit angiogenesis via enhanced secretion of SVEGFR1 independent of GM-CSF but dependent on TIMP-1 in endothelial and glioblastoma cells. *Molecular Oncology*.

[56] Ikeda Y., Hayashi I., Kamoshita E. (2004). Host stromal bradykinin B2 receptor signaling facilitates tumor-associated angiogenesis and tumor growth. *Cancer Research*.

[57] Chen Y., Yu Y., Sun S. (2016). Bradykinin promotes migration and invasion of hepatocellular carcinoma cells through TRPM7 and MMP2. *Experimental Cell Research*.

[58] Andoh T., Akira A., Saiki I., Kuraishi Y. (2010). Bradykinin increases the secretion and expression of endothelin-1 through kinin B2 receptors in melanoma cells. *Peptides*.

[59] Yang Y., Wang J., Shi F., Shan A., Xu S., Lv W. (2021). BDKRB2 is a novel EMT-related biomarker and predicts poor survival in glioma. *Aging*.

[60] Salter R. C., Ashlin T. G., Kwan A. P. L., Ramji D. P. (2010). ADAMTS proteases: key roles in atherosclerosis. *Journal of Molecular Medicine (Berlin, Germany)*.

[61] Cal S., López-Otín C. (2015). ADAMTS proteases and cancer. *Matrix Biology*.

[62] Zhao Z., Zhang K. N., Chai R. C. (2019). ADAMTSL4, a secreted glycoprotein, is a novel immune-related biomarker for primary glioblastoma multiforme. *Disease Markers*.

[63] Chen Y., Ji H., Liu S., Xing Q., Zhu B., Wang Y. (2022). Survival prognosis, tumor immune landscape, and immune responses of ADAMTS14 in clear cell renal cell carcinoma and its potential mechanisms. *Frontiers in Immunology*.

[64] Vallejo A., Perurena N., Guruceaga E. (2017). An integrative approach unveils FOSL1 as an oncogene vulnerability in KRAS-driven lung and pancreatic cancer. *Nature Communications*.

[65] Xu H., Jin X., Yuan Y. (2017). Prognostic value from integrative analysis of transcription factors c-Jun and Fra-1 in oral squamous cell carcinoma: a multicenter cohort study. *Scientific Reports*.

[66] Chen Z., Wang S., Li H. L. (2022). FOSL1 promotes proneural-to-mesenchymal transition of glioblastoma stem cells via UBC9/CYLD/NF-*κ*B axis. *Molecular Therapy*.

[67] Liu L., Yang S., Lin K. (2022). Sp1 induced gene TIMP1 is related to immune cell infiltration in glioblastoma. *Scientific Reports*.

[68] Aaberg-Jessen C., Fogh L., Sørensen M. D., Halle B., Brünner N., Kristensen B. W. (2019). Overexpression of TIMP-1 and sensitivity to topoisomerase inhibitors in glioblastoma cell lines. *Pathology & Oncology Research*.

[69] Husby G., Hoagland P. M., Strickland R. G., Williams R. C. (1976). Tissue T and B cell infiltration of primary and metastatic cancer. *The Journal of Clinical Investigation*.

[70] Liau L. M., Prins R. M., Kiertscher S. M. (2005). Dendritic cell vaccination in glioblastoma patients induces systemic and intracranial T-cell responses modulated by the local central nervous system tumor microenvironment. *Clinical Cancer Research: An Official Journal of the American Association for Cancer Research*.

[71] Authier A., Farrand K. J., Broadley K. W. R. (2015). Enhanced immunosuppression by therapy-exposed glioblastoma multiforme tumor cells. *International Journal of Cancer*.

[72] Crane A. T., Chrostek M. R., Krishna V. D. (2020). Zika virus-based immunotherapy enhances long-term survival of rodents with brain tumors through upregulation of memory T-cells. *PLoS One*.

[73] Pombo Antunes A. R., Scheyltjens I., Duerinck J., Neyns B., Movahedi K., Van Ginderachter J. A. (2020). Understanding the glioblastoma immune microenvironment as basis for the development of new immunotherapeutic strategies. *eLife*.

[74] Wang J., Matosevic S. (2019). *NT5E/CD73* as correlative factor of patient survival and natural killer cell infiltration in glioblastoma. *Journal of Clinical Medicine*.

